# Ovarian reserve in women of late reproductive age by the method of treatment of PCOS 

**Published:** 2015-05

**Authors:** Ketevan Beltadze, Ludmila Barbakadze

**Affiliations:** *Department of Reproductology, Obstetrics and Gynecology, Medical Faculty, I. Javakhishvili Tbilisi State University, Tbilisi, Georgia.*

**Keywords:** *Polycystic ovarian syndrome*, *Ovarian reserve*, *Anti*-*mullerian hormone*

## Abstract

**Background:**

The prevalence of polycystic ovarian syndrome (PCOS) particularly is increased in adolescents. Very few longitudinal follow-up for assessment of ovarian reserve in women of late reproductive age with previously confirmed PCOS have been conducted, especially after its diagnosis and treatment in adolescence.

**Objective:**

The aim of the present study was to compare of the ovarian reserve of the women of late reproductive age by the method of treatment of PCOS in adolescence.

**Materials and Methods:**

This cross sectional study in an unselected population was conducted from January to June 2014. A total of 123 women of late reproductive age were included. They had been diagnosed with PCOS between 1984 and 1990 when they were 13-18 yr. From these, first group of the study was consisted of 67 participants who underwent conservative treatment with antiandrogens and combined oral contraceptives and second group of the study was consisted of 56 participants after surgery (34-bilateral ovarian drilling and 22- ovarian wedge resection). At the time of investigation patients were 35-45 yr. The participants were collected via analysis of histories at primary diagnosis of PCOS in adolescence and at the time of the investigation analyses of reproductive hormones were conducted. Data were compared between the groups.

**Results:**

After conservative treatment PCOS women had higher levels of anti- mullerian hormone and lower follicle-stimulating hormone levels (p=0.02 and p=0.04, respectively). The number of antral follicles and mean ovarian volume were significantly greater also, than in women who underwent surgical treatment (p=0.03 and p=0.04, respectively).

**Conclusion:**

Our data suggest that PCOS patients who underwent conservative treatment have the better ovarian reserve than women who underwent surgical treatment of PCOS in adolescence.

## Introduction

Ovarian reserve is the ability of the ovary to provide egg cells that are capable of fertilization. Ovarian reserve is an important factor to predict the outcome of assisted reproductive techniques (1, 2). Variables used to estimate the ovarian reserve include age, basal or stimulated levels of follicle-stimulating hormone (FSH), estradiol (E_2_), anti-mullerian hormone (AMH), inhibin B and the number of antral follicles and ovarian volume, assessed by transvaginal ultrasound (3). Last years, serum AMH measurement has been introduced as one of the best markers of ovarian reserve (4, 5). AMH is secreted by the granulosa cells from pre-antral and antral follicles. Its main function is the inhibition of primordial follicle growth that is important in dominant follicle selection (6). 

The higher the antral follicle count, the higher the AMH levels. In Georgia, as well as around the world polycystic ovarian syndrome (PCOS) is thought to be one of the leading causes of female infertility and represents an actual problem in gynecology. It affects 4-12% of women of reproductive age and is the major factor of an ovulatory infertility (7). Its prevalence particularly is increased in adolescents (8). This population deserves attention considering the future fecundity and long term reproductive results (9). Because women with PCOS have high numbers of antral follicles, high AMH levels are often seen as well. Besides being used as a potential diagnostic marker for PCOS, AMH is used as an indicator of ovarian reserve as a predictor of ovarian response to stimulation during In vitro fertilization (IVF), that is especially important in women of late reproductive age (2, 10).

In Georgia, in 1984-1990 the surgical treatment was suggested with PCOS adolescent patients with severe clinical symptoms (severe hirsutism, acyclic bleeding). The choice of surgical treatment ovarian wedge resection or laparoscopic ovarian drilling (LOD) was depended on the clinical manifestation of PCOS. In more severe cases ovarian wedge resection was preferred. In recent years LOD is an alternative option for infertile women who have lack of response to the drug because it leads to injuries in ovarian tissue and reduces ovarian reserve. The latter is correlates with the content of ovarian tissue removed during surgery and the damage to the ovarian vascular system (11). Very few longitudinal follow-up studies for assessment of ovarian reserve in women of late reproductive age with previously confirmed PCOS have been conducted, especially after the diagnosis and treatment of PCOS in adolescence. 

In order to investigate whether surgical treatment can probably reduce ovarian reserve the aim of the present study was to compare it in women of late reproductive age after conservative treatment and surgery of PCOS in adolescence.

## Materials and methods

A total of 123 women of late reproductive age with confirmed primary PCOS in adolescence were included in this cross sectional study that was conducted from January to June 2014. All subjects underwent a clinical examination at the Archil Khomasuridze Institute of Reproductology. Informed consent was obtained from all participants and the local Committee of Ethics approved the project. They were divided into two groups: 1) after conservative treatment (antiandrogens, combined oral contraceptives) and 2) after surgical treatment (bilateral LOD or ovarian wedge resection). It should be mentioned that the surgery is not indicated in adolescence PCOS patients, but in Georgia, in 1984-1990 surgery was suggested in PCOS adolescent patients with severe clinical symptoms (severe hirsutism, acyclic bleeding).

Inclusion criteria for the study were diagnosis of PCOS between1984-1990 and at the time of original diagnosis subjects were 13-18 yr of age, at the follow-up from 35-45 years. Only patients with a diagnosis of PCOS according to the Rotterdam criteria (12) were included. In addition to the ultrasound criteria, one of the following two features had to be present for the PCOS diagnosis: 1) Oligomenorrhoea, with eight or fewer menstruations in the previous 12 months, or amenorrhea, and/or 2) Clinical and/or biochemical signs of hyperandrogenism such as testosterone 2.7 nmol/l, elevated dehydroepiandrosterone sulfate or hirsutism (8 on the Ferriman and Gallway scale). The exclusion criteria were thyroid dysfunction (normal s-TSH), adrenocortical dysfunction (normal 17-hydroxyprogesterone) or hyperprolactinaemia (prolactin <30 mg/l). Subjects without ultrasound examination were not included also. 

**Ultrasound examination**

Transvaginal ultrasound examination was undertaken on third day of the cycle. It was performed with a 7 MHz transvaginal probe. Ovarian volume was calculated in the largest ovary according to a simplified formula for an ellipsoid (0.523× length× width× thickness). All follicles, antral and growing, were counted. The presence of 12 or more follicles in each ovary measuring 2-9 mm in diameter and/or increased ovarian volume (>10 ml) was considered as PCO. Only one ovary fitting this definition was sufficient to define PCO and a dominant follicle (>10 mm) or a corpus luteum should not be present.

**Assays**

Serum concentrations of FSH, LH, E_2_ were analyzed by competitive immunoenzymatic colorimetric method for quantities determination, using commercial Nova Tec kits obtained from DiaMetra, Italy. Detection limits for the estradiol essays was 8.7 pg/mL, for FSH 0.17 mIU/ml, for LH 0.22 mIU/ml. Total coefficients of variation varied between 7.91 and 10% for these analyses. The serum concentrations of AMH were determined using enzyme linked immunoassay kits, from Immunotech Beckman Coulter Company, France. The detection limit for AMH was 0.01 ng/ml and levels below this limit were considered undetectable. Total coefficient of variation was 12.3% for the AMH analyses.

**Statistical analysis**

For comparison between groups, independent t-tests or Mann-Whitney U-tests were performed, depending on whether the variable was normally distributed or not. Frequencies were compared between groups by the χ^2^ test. The SPSS statistical package was used for all analyses (SPPS Inc. version 17.0, Chicago, IL, USA). P<0.05 was considered significant.

## Results

**Figure 1:**
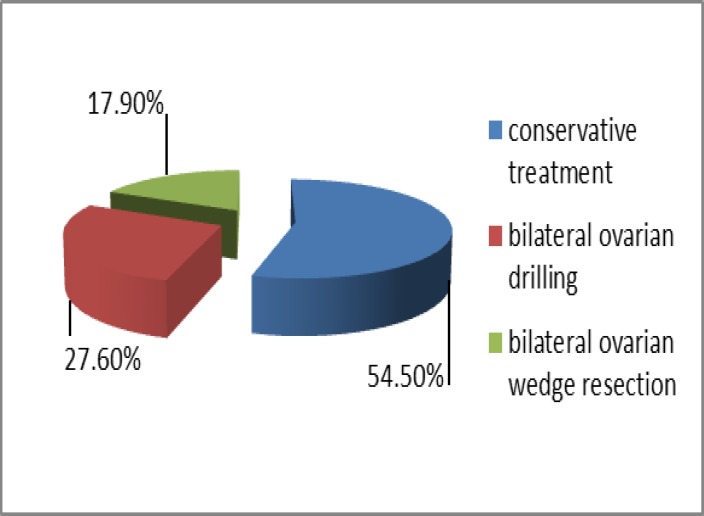
Patients according to the treatment type

A total of 123 women of late reproductive age underwent clinical examination. From these subjects from Tbilisi and regions were 92 (74.8%) and 31 (25.2%) respectively. 67 patients underwent conservative treatment and 56- surgical (34 bilateral ovarian drilling and 22 ovarian wedge resection). The patients were not infertile. Figure 1 Hence, the first study group was consisted of 67 patients who underwent conservative treatment; the second study group was consisted of 56 patients after surgical treatment. Mean ovarian volume and number of antral follicles in women of late reproductive age with history of PCOS after conservative treatment were significantly higher than after surgical treatment, 7.4±2.1 ml compared with 4.3±3.2 ml (p=0.04) and 10.5±1.4 compared with 6.1±2.7 (p=0.03), respectively (Table I). AMH serum concentrations were significantly higher in women with PCOS after conservative treatment than after surgery 3.8±2.1 compared with 1.6±1.5 (p=0.02). They had lower levels of FSH also (p=0.04) (Table I).

**Table I:** Ovarian volume, number of antral follicles, estradiol, gonadotropins and AMH in women of advanced reproductive age with a history of polycystic ovary syndrome

	**After conservative treatment (n=67)**	**After surgical treatment (n=56)**	**p-value**
Age (years)	42.7 ± 2.8	41.6 ± 3.5	0.17*
Ovarian volume (ml)	7.4 ± 2.1	4.3 ± 3.2	0.04
Antral follicle count (n)	10.5 ± 1.4	6.1 ± 2.7	0.03
Estradiol (pg/ml)	131± 16	146 ± 21	0.06*
FSH (IU/l)	7.8 ± 1.4	12.6 ± 1.1	0.04
LH (IU/l)	5.8 ± 1.8	7.4 ± 2.5	0.2*
AMH (ng/ml)	3.8 ± 2.1	1.6 ± 1.5	0.02

Data presented as mean±SD (Mann-Whitney, U-test).

* Ns: not significant

## Discussion

The previous studies showed that women with PCOS have 2-3 times higher level of the serum AMH concentration which is related to increased number of small follicles (13). Some clinical researches suggest improved fertility in ageing women with PCOS. The follicle loss through the process of ovarian ageing could explain the occurrence of more regular cycles in older patients with PCOS (5). The AMH levels in women of late reproductive age decrease (3, 14). Hence, serum AMH levels are affected by reduced number of antral follicles or ovarian tissue injuries (11). Adolescent girls with PCOS have high ovarian reserve. The question is whether surgical treatment of PCOS in adolescence affected ovarian tissue and reduced ovarian reserve in advanced age. The previous researches showed that LOD does not change AMH levels (15, 16). 

Elmashad’s research showed significant decrease AFC and AMH levels (17). This may be explained by possible damage ovarian blood vessels and ovarian tissue after electrocoagulation. The amount of ovarian tissue which is removed during surgery affects AMH produced by antral follicle (18). The surgery is not indicated in adolescence PCOS patients, but in Georgia, in 1984-1990 surgery was suggested in PCOS adolescent patients with severe clinical symptoms (severe hirsutism, acyclic bleeding). In our study women of late reproductive age had significantly reduced ovarian reserve after surgical treatment in adolescence. This could be explained by considerably damage ovarian tissue during operation, possibly considering high rate of ovarian wedge resection, which is coincided with previous data (11, 19).

Women with PCOS have lower basal FSH levels in the early follicular phase than women with normal ovaries. A lack of follicular growth may partly explain by increased production of inhibin B and high AMH levels from the increased number of antral follicles in polycystic ovaries. As ovarian ageing results in diminution of the follicular cohort in both normal women and PCOS patients, and is associated with decreased inhibin B and AMH levels, it will allow FSH enhancement and lead to full follicle maturation, more regular and ovulatory menstrual cycles (16, 20). A similar mechanism may be responsible for the increased number of ovulatory cycles after the ovarian wedge resection or ovarian drilling, when the cohort of resting follicles is abruptly reduced. Our study showed that FSH was significantly higher after surgical treatment compared with conservative that may be explained by considerably reduction of follicular cohort after surgery. 

The major limitation of the present study was the low overall response rate among PCOS patients, especially among subjects living outside the Tbilisi area. Generally a lack of researches for the evaluation of ovarian reserve after diagnosis PCOS in adolescence emphasizes the importance of the present research. It underlines that surgical treatment of PCOS in adolescence is contraindicated.

## Conclusion

The analysis of our material indicates that women of late reproductive age with history of PCOS after treatment of antiandrogens and COCs in adolescence have better ovarian reserve and possibly a good fecundity than after surgical treatment (bilateral ovarian drilling/ovarian wedge resection).
